# Magnetic Resonance Imaging Markers for Cognitive Impairment in Parkinson’s Disease: Current View

**DOI:** 10.3389/fnagi.2022.788846

**Published:** 2022-01-25

**Authors:** Yanbing Hou, Huifang Shang

**Affiliations:** Laboratory of Neurodegenerative Disorders, Department of Neurology, Rare Diseases Center, National Clinical Research Center for Geriatrics, West China Hospital, Sichuan University, Chengdu, China

**Keywords:** Parkinson’s disease, magnetic resonance imaging, biomarker, mild cognitive impairment, dementia

## Abstract

Cognitive impairment (CI) ranging from mild cognitive impairment (MCI) to dementia is a common and disturbing complication in patients with Parkinson’s disease (PD). Numerous studies have focused on neuropathological mechanisms underlying CI in PD, along with the identification of specific biomarkers for CI. Magnetic resonance imaging (MRI), a promising method, has been adopted to examine the changes in the brain and identify the candidate biomarkers associated with CI. In this review, we have summarized the potential biomarkers for CI in PD which have been identified through multi-modal MRI studies. Structural MRI technology is widely used in biomarker research. Specific patterns of gray matter atrophy are promising predictors of the evolution of CI in patients with PD. Moreover, other MRI techniques, such as MRI related to small-vessel disease, neuromelanin-sensitive MRI, quantitative susceptibility mapping, MR diffusion imaging, MRI related to cerebrovascular abnormality, resting-state functional MRI, and proton magnetic resonance spectroscopy, can provide imaging features with a good degree of prediction for CI. In the future, novel combined biomarkers should be developed using the recognized analysis tools and predictive algorithms in both cross-sectional and longitudinal studies.

## Introduction

Parkinson’s disease (PD) is becoming the fastest-growing neurological disorder. In 2016, globally, approximately 6.1 million people, suffered from PD, and this incidence is further expected to double to more than 12 million by 2040. Cardinal motor symptoms of PD are easy to identify and can be controlled by anti-PD medicine. However, various non-motor symptoms, including cognitive impairment (CI), mood disorders, neuropsychic symptoms, autonomic dysfunction, and pain, greatly aggravate the disability of the affected individuals (Dorsey et al., 2018; Collaborators, 2019; Bloem et al., 2021). CI is a common non-motor symptom that progresses from normal cognition to mild cognitive impairment (MCI), and eventually dementia. Dementia is a common disorder occurring in advanced stages of PD, and MCI, the pre-dementia stage of CI, has certainly attracted great attention in recent years. To promote uniformity in clinical and research applications on cognition in PD, clinical diagnostic criteria for different stages of CI (MCI and dementia) have been delineated by a panel of experts from the Movement Disorder Society (MDS) Task Force (Emre et al., 2007; Litvan et al., 2012).

The diagnostic criteria of PD patients with MCI (PD-MCI) are as follows: (1) a diagnosis of PD; (2) a gradual decline of the cognitive ability in the level I category based on a brief cognitive assessment or in the level II category based on a more comprehensive neuropsychological assessment including at least two tests for each of five cognitive domains (attention, executive function, language, memory, and visuospatial function); and (3) complete functional independence. Impairment in neuropsychological tests is defined per one to two standard deviations (SDs) below the appropriate norms, a significant decrease in the serial cognitive tests, or a significant decrease from the estimated premorbid levels. The PD patient with MCI according to the level II criteria shows impairment in at least two tests within one single cognitive domain or different cognitive domains (Litvan et al., 2012). Following are the diagnostic criteria of PD patients with dementia (PDD): (1) a diagnosis of PD; (2) progressive impairment in more than one cognitive domain or a significant decrease from premorbid levels; and (3) impaired functional independence (Emre et al., 2007). Based on the MDS Task Force Level II criteria, recent studies report that the frequency of PD-MCI is between 20% and 35% in newly diagnosed PD patients (Broeders et al., 2013; Pedersen et al., 2017). Moreover, according to the clinical criteria, a recent meta-analysis comprising of 41 studies reported that the pooled PD-MCI prevalence is 40% in a total sample of 7,053 PD patients (Baiano et al., 2020). In a prospective population-based study, among the newly diagnosed PD patients with normal cognition at baseline, the cumulative incidence of PD-MCI is 9.9% after 1 year, 23.2% after 3 years, and 28.9% after 5 years of follow-up (Pedersen et al., 2017). Patients with PD-MCI are more likely to eventually develop PDD, and 59.1% of patients with persistent PD-MCI within 1 year develop PDD (Pedersen et al., 2017). The point prevalence of PDD is close to 30% in a PD cohort, and at least 75% of PD patients who survived for more than 10 years are more likely to develop dementia (Aarsland and Kurz, 2010).

Although dementia is a common problem in patients with advanced PD, there are great differences in the rate of cognitive decline and the time of dementia onset in PD patients. Thus, effective markers that can accurately quantify pathological changes are needed to identify PD patients at high risk of developing CI, to predict the progression of cognitive dysfunction, and for ease of their availability. In the last few years, magnetic resonance imaging (MRI) technology, an attractive option, has improved the understanding of the pathophysiology of CI in PD patients, along with establishing potential biomarkers. These biomarkers for cognitive dysfunction in PD were reviewed based on a PubMed search for peer-reviewed articles published between January 2011 and August 2021 using the search terms “marker” OR “biomarker” AND “Parkinson’s disease” OR “Parkinson” OR “Parkinson’s disease” AND “mild cognitive impairment” OR “MCI” OR “Parkinson’s disease dementia” OR “dementia” OR “cognitive impairment” AND “MRI” OR “magnetic resonance imaging”. This review provides an update on the recent advances in the emerging role and development of MRI markers for cognitive dysfunction in PD.

## Structural Magnetic Resonance Imaging

Most of the studies in recent years acquired structural MR images using a 3.0 T scanner, and grey matter (GM) abnormalities are extensively examined using either voxel-based morphometry (VBM) or surface-based analyses. Automated and unbiased VBM analysis can be used to estimate the GM volume or concentration differences between groups at the whole-brain level, thus, it has been widely used to quantify regional GM alterations in PD patients with CI. Cortical thickness, the distance between white and pial surfaces, is a more sensitive structural MRI-based measurement to detect early GM changes. The details of imaging methods are presented in Table 1. The region of interest (ROI) approach has been used to analyze both cortical and subcortical changes based on an *a priori* hypothesis. Summary findings from structural MRI correlated with cognitive dysfunction in PD are showed in Table 2.

**TABLE 1:** Imaging characteristics of magnetic resonance imaging studies in Parkinson’s disease and cognitive impairment.

Modality	MR sequence	Core software/method	Analysis variables	Advantages/disadvantages
Structural imaging	T1-TFE sequence/3D SPGR/MPRAGE	SPM (http://www.fil.ion.ucl.ac.uk/spm)/ Freesurfer (http://surfer.nmr.mgh.harvard.edu)	Voxel-based morphometry (VBM)	It can estimate the gray matter (GM) volume or concentration alterations at the whole-brain level.
		Freesurfer (http://surfer.nmr.mgh.harvard.edu)	Cortical thickness (CTh)	A more sensitive structural MRI-based measurement to detect early GM changes.
MRI related to small-vessel disease	T2-weighted fast spin-echo sequence/FLAIR	Fazekas visual rating scale (Scheltens et al., 1993)	White matter hyperintensity (WMH)	It can reflect pathological white matter (WM) tissue ischemia. It can be assessed on the basis of size and quantity of lesions in a given neuroanatomical location.
		Automated routines (de Boer et al., 2009; Bohnen and Albin, 2011)	WMH	Quantitative assessment of WML burden may be particularly advantageous for assessing changes over time compared with the visual grading.
		Standards for Reporting Vascular Changes on Neuroimaging criteria (Wardlaw et al., 2013)	Perivascular space (PVS)	It is called the Virchow-Robin spaces and is piallined interstitial fluid-filled space surrounding the penetrating vessels.
Neuromelanin-sensitive MRI	T1-weighted FSE sequence	Semi-automated analysis (Sulzer et al., 2018).	Signal intensity	It can depict characteristics of tissue containing neuromelanin.
Quantitative susceptibility mapping (QSM)	3D-fast low-angle shot sequence/3D fast-field echo sequence	Matlab (MathWorks, Natick, MA, United States)/Voxel-based QSM analysis	QSM values	It can quantify susceptibility-changing materials, such as iron accumulation.
Diffusion tensor imaging	Pulsed gradient spin-echo single- shot echo-planar DT MR imaging sequences	FSL Diffusion Toolbox (FMRIB Software Library, Center Software Library, University of Oxford, Oxford, United Kingdom);	Fractional anisotropy (FA)/mean diffusivity (MD)	The directionality of movement, FA, and its total magnitude, MD, both can sensitively detect early microstructural cortical damage.
MRI related to cerebrovascular abnormality	cASL/pASL	ANTs (http://stnava.github.io/ANTs)	Cerebral blood flow (CBF) values	Cerebrovascular abnormalities include altered CBF, cerebral blood volume (CBV), and blood–brain barrier permeability.
	iVASO MRI	Matlab (MathWorks, Natick, MA, United States)/SPM (http://www.fil.ion.ucl.ac.uk/spm)	Arteriolar CBV (CBVa) values	The measurement of changes in CBVa may be more sensitive than measurement of changes in total CBV and CBF.
Resting-state functional MRI	Functional gradient-echo EPI sequence	SPM (http://www.fil.ion.ucl.ac.uk/spm) FSL (http://www.fmrib.ox.ac.uk/fsl/)	Functional connectivity	It can measure intrinsic blood oxygen level-dependent (BOLD) low-frequency signal fluctuations.
Proton magnetic resonance spectroscopy (^1^H-MRS)	PRESS sequences	Matlab (MathWorks, Natick, MA, United States)/SPM (http://www.fil.ion.ucl.ac.uk/spm)	Average MRS signals	It can reflect the integrity of different elements in the brain, including *N*-acetylaspartate (NAA), choline compounds (Cho), creatine (Cr), and myo-inositol (MI).

*cASL, continuous arterial spin labeling; EPI, echo-planar imaging; FLAIR, fluid-attenuated inversion-recovery; FSE, fast spin-echo; iVASO, inflow-based vascular-space-occupancy; MPRAGE, magnetization prepared rapid gradient echo; pASL, pulsed arterial spin labeling; PRESS, point resolved spectroscopy; SPGR, spoiled gradient echo; TFE, turbo field echo.*

**TABLE 2:** Summary findings from structural magnetic resonance imaging studies in Parkinson’s disease and cognitive impairment.

Studies		Results
Cross-sectional	Whole brain analysis: Voxel-based meta-analysis/coordinate-based meta-analysis;	**PD-MCI vs. PDNCI:** Gray matter (GM) atrophy in left superior frontal gyrus (SFG), left superior temporal lobe (STL), and left insula (Xu et al., 2016). GM atrophy in bilateral dorsolateral prefrontal cortex, left angular gyrus, right supramarginal gyrus, left insula, and midcingulate cortex (Mihaescu et al., 2019). GM atrophy in the left anterior insula extending to the inferior frontal gyrus, and orbital part (Zheng et al., 2019).
	Voxel-based meta-analysis/coordinate-based meta-analysis;	**PDD vs. PDND:** GM atrophy in the left SFG, bilateral STL extending to hippocampus (Xu et al., 2016). GM atrophy in bilateral insula and right hippocampus (Mihaescu et al., 2019).
		**Across various status of cognitive decline:** A pattern of linear and progressive cortical changes (reduced GM volume or cortical thinning) (Song et al., 2011; Melzer et al., 2012; Pagonabarraga et al., 2013).
	Subcortical and region of interest analyses:	**PD-MCI vs. PDNCI:** Atrophy in hippocampal subregions [cornu ammonis 1 (CA1) and hippocampal-amygdaloid transition area (HATA)] (Becker et al., 2021), entorhinal cortex (ENT) (Goldman et al., 2012; Jia et al., 2019), amygdala (Schulz et al., 2018), thalamus (Mak et al., 2014; Chen et al., 2016; Schulz et al., 2018), accumbens nucleus (Mak et al., 2014), and nucleus basalis of Meynert (Schulz et al., 2018).
		**Across various status of cognitive decline:** A progressive decrease in volume of the hippocampus and substantia innominata (SI) (Weintraub et al., 2011; Choi et al., 2012).
Longitudinal	Whole brain analysis:	**PD-MCI vs. PDNCI:** Increased global atrophy (Mak et al., 2017). A higher rate of cortical thinning in frontal/supplementary motor area, parietal-temporal, and occipital cortices (Hanganu et al., 2014; Mak et al., 2015).
	Subcortical and region of interest analyses:	**PD-MCI vs. PDNCI:** A greater progression of cortical thinning in the posterior regions of brain (Garcia-Diaz et al., 2018). A decreased volume in the amygdala and accumbens nucleus (Hanganu et al., 2014; Mak et al., 2015).
	Converters versus non-converters:	**PD-MCI converters vs. non-converters:** GM atrophy in temporal regions and the amygdala/hippocampus structural covariance network at baseline (Wen et al., 2015; Zhou et al., 2020; Chen et al., 2021). Cortical thinning in the anterior cingulate cortex, parietal, temporal, and occipital regions, and GM atrophy in accumbens nucleus at baseline (Mak et al., 2015; Foo et al., 2017a,b; Filippi et al., 2020; Gorges et al., 2020). A higher progression of atrophy in the frontal lobe and longitudinal white matter (WM) volumetric reduction (Wen et al., 2015; Zhou et al., 2020; Chen et al., 2021). A higher progression of atrophy in the thalamus, caudate nucleus, accumbens nucleus and hippocampal subfields (CA2-3) (Mak et al., 2015; Foo et al., 2017a,b; Filippi et al., 2020; Gorges et al., 2020).
		**PDD converters vs. non-converters:** GM atrophy in uncus, global hippocampus, and hippocampal subfields (presubiculum, subiculum, and CA1) at baseline (Gee et al., 2017; Low et al., 2019). Larger ventricular volume at baseline (Camicioli et al., 2011). Cortical thinning in the precentral gyrus and superior frontal gyrus (RSFG) and anterior cingulate at baseline (Compta et al., 2013). A higher progression of atrophy in the global hippocampus, hippocampal subfields [parasubiculum, presubiculum, granule cell layer of the dentate gyrus (GC-DG), and fimbria] and parahippocampal gyrus (Gee et al., 2017; Low et al., 2019). **PDD converters vs. PD-MCI non-converters:** GM atrophy in prefrontal areas, insular and caudate nucleus, smaller normalized SI volume, and cortical thinning extending from the posterior cortical area into the frontal region, and the frontotemporal cortices (Lee et al., 2014; Chung et al., 2019; Filippi et al., 2020).
	Predictive variables for conversion	**Predictors for PD-MCI conversion:** Baseline volumes of global WM, global hippocampus, and hippocampal subregions (parasubiculum, CA4, GC-DG, and HATA), thalamus, and accumbens nucleus (Kandiah et al., 2014; Wen et al., 2015; Foo et al., 2017a,b).
		**Predictors for PDD conversion:** Baseline volumes of global hippocampus and hippocampal subregions (presubiculum, subiculum, and CA1), cortical thickness of the medial superior frontal/olfactory cortices, superior frontal/anterior cingulate and precentral regions (Compta et al., 2013; Kandiah et al., 2014; Chung et al., 2019; Low et al., 2019).
		**Predictors for CI conversion:** A validated AD-spatial pattern of brain atrophy (Tropea et al., 2018). GM volume in the nucleus basalis of Meynert (Ray et al., 2018; Schulz et al., 2018).

*CI, cognitive impairment; MCI, mild cognitive impairment; PD, Parkinson’s disease; PDD, PD patients with dementia; PD-NCI, PD without CI; PD-ND, PD without dementia.*

### Cross-Sectional MR Studies

Cross-sectional results on CI-related GM changes in PD patients remain heterogeneous, and three meta-analyses were conducted to identify the most consistent GM alterations associated with PD-CI (Xu et al., 2016; Mihaescu et al., 2019; Zheng et al., 2019). A meta-analysis study in 2016, using five studies compared 92 PD-MCI patients with 192 PD without CI (PD-NCI) patients, and from ten studies, compared 168 PDD patients with 233 PD without dementia (PD-ND) patients. The authors have reported significantly reduced GM volume in the left superior frontal gyrus (SFG), left superior temporal lobe (STL), and left insula in the PD-MCI group, and in the left SFG, and bilateral STL extending to the hippocampus in the PDD group (Xu et al., 2016). A meta-analysis study in 2019 compared 405 PD-MCI patients with 559 PD-NCI patients from fifteen studies, and compared 178 PDD patients with 278 PD-ND patients from eleven studies. A significantly higher GM atrophy in bilateral dorsolateral prefrontal cortex, left angular gyrus, right supramarginal gyrus, left insula, and midcingulate cortex in the PD-MCI group, and bilateral insula and right hippocampus in the PDD group was reported (Mihaescu et al., 2019). Another meta-analysis study in 2019 consisting of 504 PD-MCI patients and 554 PD-NCI patients from twenty studies reported a drastically remarkable GM atrophy in the left anterior insula extending to the inferior frontal gyrus, and orbital part in the PD-MCI group (Zheng et al., 2019). GM atrophy in the insula and frontal-limbic-temporal regions are robust features of CI in PD, and unilateral-to-bilateral development of structural atrophy is a potential indicator for progression of PD-MCI to PDD. In particular, several studies attempted to analyze GM changes across various stages of cognitive decline by comparing a prospective sample of PD-NCI, PD-MCI, PDD patients, and healthy subjects (HCs). These typically report a pattern of linear and progressive cortical changes (reduced GM volume or cortical thinning) with increasing levels of CI (Song et al., 2011; Melzer et al., 2012; Pagonabarraga et al., 2013).

Notably, several structural MRI studies focused on assessing the differences in subcortical GM structures or ROIs crucial in the cognitive functions of PD patients. The volume of the hippocampus and substantia innominata (SI), a major source of cholinergic input to the cerebral cortex, progressively decreases corresponding to the cognitive status (Weintraub et al., 2011; Choi et al., 2012). Recent advances in the imaging technology and software analysis tools have made the study of hippocampal subregions *in vivo* a possibility (Iglesias et al., 2015). Smaller cornu ammonis 1 (CA1) and hippocampal-amygdaloid transition area (HATA) volumes have been observed in PD-MCI patients relative to the PD-NCI patients (Becker et al., 2021). In addition, as compared with the PD-NCI group, the PD-CI group exclusively exhibits atrophy in the entorhinal cortex (ENT) (Goldman et al., 2012; Jia et al., 2019), amygdala (Schulz et al., 2018), thalamus (Mak et al., 2014; Chen et al., 2016; Schulz et al., 2018), accumbens nucleus (Mak et al., 2014), and nucleus basalis of Meynert (Schulz et al., 2018).

### Longitudinal MR Studies

Recent longitudinal studies described the progression patterns of GM changes in PD patients with CI, which can facilitate a better understanding of the mechanism of CI in PD. Patients with PD-MCI show increased global atrophy within 18 months relative to both PD-NCI patients and HCs (Mak et al., 2017). Other studies have also attempted to investigate the longitudinal changes in the cortical and subcortical GM and reported a higher rate of cortical thinning in the frontal/supplementary motor area, parietal-temporal, occipital cortices and a significant decrease in the volume of the amygdala and accumbens nucleus in PD-MCI patients relative to both PD-NCI patients and HCs (Hanganu et al., 2014; Mak et al., 2015). Some longitudinal studies have examined the differential progressive GM loss in the specific cortical regions. A 4-year follow-up study reported that over time, as compared to the PD-NCI patients, those with PD-MCI had significantly greater progression of cortical thinning in the posterior regions of the brain related to visuospatial and visuoperceptual (VS/VP) changes (Garcia-Diaz et al., 2018). Another 4-year follow-up study held the opinion that the hippocampal subfields have different vulnerabilities to the degenerative processes related to aging as well as PD. PD patients and HCs have similar longitudinal changes in CA1, while both PD-NCI and PD-MCI patients have a more severe decline in the anterior and posterior hippocampal segments, implying its predictive value for assessing memory dysfunction (Uribe et al., 2018).

### Converters Versus Non-converters

Researchers have examined structural alterations in the brain to predict cognitive decline in PD patients. PD patients with normal cognition at baseline and who develop PD-MCI during follow-up are classified as “PD-MCI converters,” while those who remain cognitively asymptomatic during the follow-up are classified as “non-converters.” Relative to the non-converters, PD-MCI converters have a significant reduction in GM volume of temporal regions and the amygdala/hippocampus structural covariance network at baseline, along with evidently progressive frontal lobe atrophy and longitudinal white matter (WM) volumetric reduction (Wen et al., 2015; Zhou et al., 2020; Chen et al., 2021). Some studies have further investigated the pattern and progression of cortical thickness and subcortical volumes in PD-MCI converters, and reported a higher degree of anterior cingulate cortex, parietal, temporal, and occipital cortices thinning, atrophy of accumbens nucleus at baseline, and progressive atrophy in the thalamus, caudate nucleus, accumbens nucleus, and CA2-3 of hippocampal subregions (Mak et al., 2015; Foo et al., 2017a,b; Filippi et al., 2020; Gorges et al., 2020).

Non-demented PD patients who progress to PDD during follow-up are classified as “PDD converters.” At baseline, relative to the non-converters, PDD converters have lower GM volume in uncus, global hippocampus, and hippocampal subfields (presubiculum, subiculum, and CA1) (Gee et al., 2017; Low et al., 2019), lesser thicknesses of the precentral gyrus and superior frontal gyrus (RSFG) and anterior cingulate (Compta et al., 2013), and larger ventricular volume (Camicioli et al., 2011). Moreover, over time, PDD converters have higher GM atrophy in the global hippocampus, hippocampal subfields [parasubiculum, presubiculum, granule cell layer of the dentate gyrus (GC-DG), and fimbria] and parahippocampal gyrus (Gee et al., 2017; Low et al., 2019). Another study classified PD-MCI patients as PDD converters or PD-MCI non-converters based on whether they are subsequently diagnosed with PDD during follow-up. Relative to PD-MCI non-converters, the PDD converters were found to have lower GM density in prefrontal areas, insular and caudate nucleus, smaller normalized SI volume, and a lesser cortical thickness extending from the posterior cortical area into the frontal region, and the frontotemporal cortices (Lee et al., 2014; Chung et al., 2019; Filippi et al., 2020).

### Predictive Variables for Conversion

Baseline volumes of global WM, global hippocampus, and hippocampal subregions (parasubiculum, CA4, GC-DG, and HATA), thalamus, and accumbens nucleus are significant neuroimaging predictors for determining the conversion from PD-NCI to PD-MCI; the baseline global GM volume cannot significantly predict the conversion to PD-MCI (Kandiah et al., 2014; Wen et al., 2015; Foo et al., 2017a,b). Moreover, GM volume in the global hippocampus and hippocampal subregions (presubiculum, subiculum, and CA1), cortical thickness in the medial superior frontal/olfactory cortices, superior frontal/anterior cingulate and precentral regions are significant predictors for the progression to PDD (Compta et al., 2013; Kandiah et al., 2014; Chung et al., 2019; Low et al., 2019).

Growing evidence highlighted that brain regions undergoing neurodegeneration may overlap synergistically with the pathological processes for PD and Alzheimer’s disease (AD) (Masliah et al., 2001; Clinton et al., 2010). To determine its association with CI, a validated AD-spatial pattern of brain atrophy (spatial pattern of abnormality for recognition of early AD index, SPARE-AD) was quantified in PD patients with a range of cognitive abilities. The findings support the involvement of hippocampus and parietal-temporal cortex with CI in PD (Weintraub et al., 2012), and affirm the importance of AD biomarkers as effective predictors for CI in PD (Tropea et al., 2018). In addition, autopsies have identified degeneration of the cholinergic basal forebrain, especially in the nucleus basalis of Meynert, causing cortical cholinergic depletion, and eventually leading to the CI in patients with PD and AD (Liu et al., 2015). GM volume in the nucleus basalis of Meynert was a critical cholinergic biomarker to predict CI in PD-NCI patients (Ray et al., 2018; Schulz et al., 2018).

## Other Magnetic Resonance Imaging Techniques

### Magnetic Resonance Imaging Related to Small-Vessel Disease

As a consequence of cerebral small-vessel disease (SVD), white matter hyperintensities (WMHs) in the brain can reflect pathological WM tissue ischemia (Erten-Lyons et al., 2013). PD patients with greater WMH have more severe cognitive deficits and a higher annual rate of change in global cognition (Dadar et al., 2018; Chahine et al., 2019; Chang et al., 2021). The periventricular WMH burden is significantly associated with PD-MCI and severe WMH with PDD; both are independent of cardiovascular risk factors (Huang et al., 2020; Tsai et al., 2021). In addition, heavier temporal WMH burden is associated with worse verbal memory (Chahine et al., 2019), and higher periventricular WMH burden correlates with greater decline in the executive and visuospatial functions (Huang et al., 2020). Therefore, WMH is strongly correlated with CI in PD and may serve as a surrogate marker for CI in PD (Kandiah et al., 2014; Fang et al., 2021). Visible perivascular space (PVS) has also been considered as an imaging biomarker for SVD. A longitudinal MRI-visible PVS study reported higher PVS severity in basal ganglia (BG) as a predictor of cognitive conversion of PD-NCI patients as well as for cognitive decline in PD-MCI patients (Park et al., 2019). Summary findings from other MRI techniques correlated with cognitive dysfunction in PD can be found in Table 3.

**TABLE 3:** Summary findings from other magnetic resonance imaging studies in Parkinson’s disease and cognitive impairment.

Modalities	Results	
MRI related to small-vessel disease	White matter hyperintensity (WMH)	PD patients with greater WMH have more severe cognitive deficits and a higher annual rate of change in global cognition (Dadar et al., 2018; Chahine et al., 2019; Chang et al., 2021). The periventricular WMH burden is significantly associated with PD-MCI and severe WMH with PDD (Huang et al., 2020; Tsai et al., 2021). WMH is strongly correlated with CI in PD and may serve as a surrogate marker for CI in PD (Kandiah et al., 2014; Fang et al., 2021).
	Perivascular space (PVS)	Higher PVS severity in basal ganglia (BG) as a predictor of cognitive conversion of PD-NCI patients as well as for cognitive decline in PD-MCI patients (Park et al., 2019).
Neuromelanin-sensitive MRI (NM-MRI)	Signal intensity	**PD-MCI vs. HC** Lower NM signal intensity in the locus coeruleus, which may serve as an imaging marker for assessing cognitive dysfunction in PD (Li et al., 2019; Prasuhn et al., 2021).
Quantitative susceptibility mapping (QSM)	QSM values	**PD-MCI vs. PDNCI** Higher QSM values in the orbitofrontal cortex, cuneus, precuneus, fusiform gyrus, and head of the caudate nucleus (Uchida et al., 2019). **PDD vs. PDND** Higher QSM values in the left hippocampus (Li et al., 2018).
Diffusion tensor imaging	Fractional anisotropy (FA)/mean diffusivity (MD)	**PD-MCI vs. PDNCI** Decreased FA values in the anterior and superior corona radiata, genu, and body of the corpus callosum, anterior inferior fronto-occipital fasciculus (IFOF)/uncinate, and anterior superior longitudinal fasciculi (SLF), frontotemporoparietal regions, hippocampus, BG, amygdala, thalamus (Agosta et al., 2014; Galantucci et al., 2017; Schulz et al., 2018). Increased MD values in hippocampus, entorhinal cortex (ENT), amygdala, thalamus, and nucleus basalis of Meynert (Galantucci et al., 2017; Schulz et al., 2018).
		**PDD vs. PDND** Decreased FA values in the prefrontal white matter, hippocampus, and the genu of the corpus callosum (Kamagata et al., 2013; Chen et al., 2015).
		**Across various status of cognitive decline** The loss of microstructural WM integrity was spatially restricted in PD-NCI patients, while these alterations increase with progressive cognitive deterioration (Hattori et al., 2012; Deng et al., 2013; Melzer et al., 2013).
		**Predictors for CI conversion** The increased MD values in the nucleus basalis of Meynert (Schulz et al., 2018).
MRI related to cerebrovascular abnormality	Cerebral blood flow (CBF) values	**PD-MCI vs. HC** A pattern of decreased CBF at the posterior part of the brain, mostly in the lateral and medial parietooccipital cortices, summarized as “posterior hypoperfusion” (Arslan et al., 2020). Parietal CBF may serve as a potential early biomarker for CI in PD (Pelizzari et al., 2020).
	Arteriolar cerebral blood volume (CBVa) values	**PD-CI vs. HC** A decreasing trend of CBVa in the substantia nigra, caudate nucleus and putamen, and an increasing trend of CBVa in the pre-supplementary motor area and intracalcarine gyrus (Paez et al., 2020).
Resting-state functional MRI	Functional connectivity (FC)	**PD-CI vs. PDNCI** Reduced FC in specific brain regions that are parts of the default mode network (DMN) (Wolters et al., 2019).
		**Predictors for CI conversion** FC between the medial prefrontal cortex (mPFC) and posterior cingulate cortex (PCC) within DMN at baseline (Zarifkar et al., 2021).
Proton magnetic resonance spectroscopy (^1^H-MRS)	Average MRS signals	**PD-CI vs. PDNCI** Higher the choline to creatine (Cho/Cr) ratio in the occipital lobe, reduced concentration of *N*-acetylaspartate (NAA) in the middle and superior frontal gray matter regions, and reduced concentration of total Cho and total Cr in the frontal white matter regions (Nie et al., 2013; Chaudhary et al., 2021).
		Levels of NAA reduced in the dorsolateral prefrontal cortex in the early stage, and progression to the hippocampus in PDD patients (Pagonabarraga et al., 2012). Frontal neuro-metabolism may serve as a promising biomarker for characterizing cognitive deficits in patients with PD (Chaudhary et al., 2021).

*CI, cognitive impairment; MCI, mild cognitive impairment; PD, Parkinson’s disease; PDD, PD patients with dementia; PD-NCI, PD without CI; PD-ND, PD without dementia.*

### Neuromelanin-Sensitive Magnetic Resonance Imaging

Neuromelanin-sensitive MRI (NM-MRI) is a promising technique that depicts characteristics of tissue containing neuromelanin, a dark polymer product of noradrenaline metabolism (Nakamura and Sugaya, 2014). The substantia nigra (SN) of the midbrain and the locus coeruleus (LC) within the pons contain large clusters of neuromelanin-producing cells, and the loss of these neuromelanin-containing cells within the two regions is a primary pathological diagnostic criterion for PD. The NM signal intensity of the LC in PD-MCI patients is significantly lower than that of the control subjects, and may serve as an imaging marker for assessing cognitive dysfunction in PD (Li et al., 2019; Prasuhn et al., 2021).

### Quantitative Susceptibility Mapping

Quantitative susceptibility mapping (QSM) is another new MRI technique that enables the quantification of susceptibility-changing materials. Iron accumulation has been proposed as a significant pathomechanism for PD, which can be systematically examined using the QSM method. PD-MCI patients have significantly higher QSM values in the orbitofrontal cortex, cuneus, precuneus, fusiform gyrus, and head of the caudate nucleus relative to the PD-NCI patients (Uchida et al., 2019). Meanwhile, PDD patients have significantly higher QSM values in the left hippocampus as compared to non-demented PD patients (Li et al., 2018). These findings suggest the association between CI in PD and cerebral iron burden, which may be an auxiliary biomarker for early detection of CI in PD.

### MR Diffusion Imaging

Diffusion tensor imaging (DTI) is an *in vivo* surrogate measure for the microstructural integrity of WM tracts. Using this technique, the rate of 3-dimensional water molecule diffusion through a pulsed-field gradient can be measured. The directionality of movement is quantified as fractional anisotropy (FA) and its total magnitude is measured as mean diffusivity (MD). The decrease in FA and increase in MD within the central nervous system have been proposed as sensitive imaging indicators for detecting early microstructural cortical damage in patients with neurodegenerative disorders.

The PD-MCI patients have a distributed pattern of WM abnormalities in the anterior and superior corona radiata, genu, and body of the corpus callosum, anterior inferior fronto-occipital, uncinate, and superior longitudinal fasciculi relative to PD-NCI patients and HCs (Agosta et al., 2014). Lower FA values in frontotemporoparietal regions, hippocampus, amygdala, thalamus, BG, and higher MD values in hippocampus, ENT, amygdala, thalamus, and nucleus basalis of Meynert relative to PD-NCI patients have been reported in PD-MCI patients (Galantucci et al., 2017; Schulz et al., 2018). PDD patients have significantly lower FA values in the prefrontal WM, hippocampus, and the genu of the corpus callosum relative to the non-demented PD patients (Kamagata et al., 2013; Chen et al., 2015), and lower FA values in both the anterior and the posterior cingulate fiber tracts as compared to HCs (Kamagata et al., 2012). Microstructural abnormalities in WM of PD patients with variant cognitive statuses (PD-NCI, PD-MCI, and PDD) have been investigated to elucidate the pathological processes of CI in PD. The loss of microstructural WM integrity is spatially restricted in PD-NCI patients, while these alterations increase with progressive cognitive deterioration (Hattori et al., 2012; Deng et al., 2013; Melzer et al., 2013).

In a previous study, dynamic MD changes were specifically assessed by a longitudinal approach in newly-diagnosed drug-naïve PD patients. A higher 1-year MD increase in frontal and occipital cortices was found in PD patients relative to HCs, which correlated with changes in the cognitive measures (Sampedro et al., 2019). Moreover, the increased MD values in the nucleus basalis of Meynert can predict the conversion from PD-NCI to PD with CI (Schulz et al., 2018). Thus, further longitudinal investigations are required to establish the clinical utility of DTI as a sensitive biomarker for the pathology of CI in PD.

### Magnetic Resonance Imaging Related to Cerebrovascular Abnormality

Emerging evidence supported that cerebrovascular abnormalities including altered cerebral blood flow (CBF), cerebral blood volume (CBV), and blood–brain barrier permeability are linked with pathophysiology in CI. Using the arterial spin labeling (ASL) technique, brain perfusion can be quantitatively assessed in terms of CBF. A pattern of decreased CBF at the posterior part of the brain, mostly in the lateral and medial parietooccipital cortices, summarized as “posterior hypoperfusion,” has been reported in the PD-MCI group relative to HCs (Arslan et al., 2020), which may be a suitable early biomarker for CI in PD. Parietal CBF correlates to visuomotor skills and also may serve as a potential early biomarker for CI in PD (Pelizzari et al., 2020). Using the inflow-based vascular-space-occupancy (iVASO) MRI approach, potential arteriolar CBV (CBVa) abnormalities reflecting the homeostasis of the most actively regulated segment in the microvasculature can be calculated. A decreasing trend of CBVa in the substantia nigra, caudate nucleus and putamen, and an increasing trend of CBVa in the pre-supplementary motor area and intracalcarine gyrus have been reported in PD-CI patients relative to HCs (Paez et al., 2020). These exploratory findings provide important information for efforts toward developing potential biomarkers for the risk of CI in PD.

### Resting-State Functional Magnetic Resonance Imaging

Resting-state functional MRI (rs-fMRI) can provide a reliable and reproducible tool to examine the functional activity in different brain networks by measuring intrinsic blood oxygen level-dependent (BOLD) low-frequency signal fluctuations. There is a growing interest in using fMRI to investigate the neural basis for CI in PD.

A voxel-based meta-analysis consisting of 289 PD-NCI, 222 PD-MCI, 68 PDD, and 353 HC subjects from seventeen studies showed that PD patients with CI had reduced functional connectivity (FC) in specific brain regions that are parts of the default mode network (DMN) (Wolters et al., 2019). Moreover, notably, increased FC may be the initial manifestation of altered brain function preceding cognitive deficits (transition from PD-NCI to PD-MCI), and these functional disruptions may be considerably associated with a cognitive decline in the progression of PD-related pathology (Gorges et al., 2015; Zhan et al., 2018; Zarifkar et al., 2021). A novel graph-theoretical analysis of functional networks indicates that the topological properties of brain networks are disrupted in PD-MCI patients with reduced long-range connections and enhanced local interconnectedness (Baggio et al., 2014). Moreover, a recent study suggested that PD-MCI patients had an increased random organization of brain networks and altered nodal centralities as compared with HCs (Hou et al., 2020). These results provide a new perspective on the functional basis of CI in PD.

In an exploratory longitudinal study, FC between the medial prefrontal cortex (mPFC) and posterior cingulate cortex (PCC) within DMN at baseline were found to be the best putative predictor for CI in PD (Zarifkar et al., 2021). PD patients with a longitudinal decline in cognitive function have a significant decline in dorsal attention-frontoparietal internetwork (DAN-FPN) strength, which may serve as a prognostic marker for assessing the development of dementia in PD (Campbell et al., 2020).

### Proton Magnetic Resonance Spectroscopy

Proton magnetic resonance spectroscopy (^1^H-MRS) owing to a high natural abundance and magnetic sensitivity of protons in the brain allows for *in vivo* measurements of four major metabolites, that reflect the integrity of different elements in the brain, including *N*-acetylaspartate (NAA), choline compounds (Cho), creatine (Cr), and myo-inositol (MI) (Nie et al., 2013).

The PD-CI patients have higher the choline to creatine (Cho/Cr) ratio in the occipital lobe, reduced concentration of NAA in the middle and superior frontal gray matter regions, and reduced concentration of total Cho and total Cr in the frontal white matter regions relative to PD-CN patients and HCs (Nie et al., 2013; Chaudhary et al., 2021). According to the differential status of CI, levels of NAA in the dorsolateral prefrontal cortex are reduced in the early stage with progression to the hippocampus in PDD patients (Pagonabarraga et al., 2012). Frontal neuro-metabolism may serve as a promising biomarker for characterizing cognitive deficits in patients with PD (Chaudhary et al., 2021).

## Conclusion

In summary, specific patterns of cognitive neural substrates in PD can be detected using various MRI techniques, and this has become a putative prediction tool for CI in PD. With the advances in image acquisition and analysis technologies, predictors derived from structural MRI are promising for assessing the cognitive dysfunction in PD. Recently, abnormal patterns from other MRI techniques also have emerged as the candidate biomarkers for evaluating the progression of cognitive decline in PD. Given that biomarkers from a single modal MRI cannot completely describe the complexity of pathological substrates of cognitive deficits in PD, we believe that the potential predictors from multi-modal MRI technology using recognized analysis tools and predictive algorithms deserve further investigation. Furthermore, targeted biomarkers for different cognitive domains and the determination of the best combination scheme of biomarkers from multi-modal MRI are the most important issues in the future.

## Author Contributions

YH prepared the initial manuscript. HS provided content expertise and reviewed the manuscript. Both authors contributed to the article and approved the submitted version.

## Conflict of Interest

The authors declare that the research was conducted in the absence of any commercial or financial relationships that could be construed as a potential conflict of interest.

## Publisher’s Note

All claims expressed in this article are solely those of the authors and do not necessarily represent those of their affiliated organizations, or those of the publisher, the editors and the reviewers. Any product that may be evaluated in this article, or claim that may be made by its manufacturer, is not guaranteed or endorsed by the publisher.
